# A longitudinal study on the effect of labor values on benign/malicious envy: the mindfulness reperceiving model

**DOI:** 10.1038/s41598-024-54504-z

**Published:** 2024-03-20

**Authors:** Qingji Zhang, Xiaomei Chao, Yeman Tu, Shunyu Yao, Peng Quan

**Affiliations:** 1 School of Education (Teachers College), Guangzhou University, Guangzhou, China; 2 School of Marxism, Dongguan University of Technology, Dongguan, China; 3 School of Elementary Education, Changsha Normal University, Changsha, China; 4 School of Chemical Engineering and Energy Technology, Dongguan University of Technology, Dongguan, China; 5 Guangzhou No.13 Middle School, Guangzhou, China; 6 School of Humanities and Management, Guangdong Medical University, Dongguan, China

**Keywords:** Labor values, Benign envy, Malicious envy, Mindfulness, Longitudinal study, Psychology, Human behaviour

## Abstract

This study investigates the relationship between labor values and two forms of envy—benign and malicious—as well as the potential mediating role of mindfulness using a mindfulness reperceiving model. Two thousand three hundred sixty three Chinese teenagers participated in a longitudinal study over an eight-month period, completing questionnaires measuring labor values, benign envy, malicious envy, and mindfulness. The cross-sectional data showed that labor values had an immediate negative effect on malicious envy, with mindfulness partially mediating this relationship. Additionally, labor values had an immediate positive effect on benign envy, but mindfulness did not mediate this relationship. Longitudinal data analysis revealed that the delayed effect of labor values on later benign/malicious envy was similar to its immediate effect. However, mindfulness only played a mediating role in the relationship between labor values and later malicious envy. Cross-gender stability was found in both the immediate effect model and the delayed effect model. Overall, this study sheds light on the influence of labor values on the development of social emotions and the potential mediating role of mindfulness in the Chinese cultural context.

## Introduction

Values refer to the belief systems we use to judge right and wrong, good and evil, and gain and loss, which not only guide us in terms of pursuing our own ideals, but also influence our life choices. Values can be distinct from other various perspectives and influences, one of which is labor values, which build their pragmatic content based on life practice and reflect our views and emotional attitudes towards the fundamental value of labor. Meanwhile envy, which is thought to be a common social emotion, may be influenced not only by one’s own personal values, but also by labor values. Therefore, this study aimed to explore the influence of labor values on envy, or more specifically, how labor values affect the two subtypes of envy—benign and malicious—and the internal mechanism involved from the perspective of mindfulness reperceiving model using a longitudinal study design.

Envy is defined as a type of negative emotion caused by upward social comparison^1,2^. Recent studies have found that envy is in fact multifaceted and can be construed in either as relatively positive (i.e., benign envy) or relatively negative (i.e., malicious envy)^3–6^. van de Ven, Zeelenberg^4^ found that benign envy is more likely to be elicited when the advantages experienced by the subject of one’s envy are derserved, while malicious envy is elicited when one’s advantages are undeserved. Benign envy may indeed motivate individuals to learn from the envied, while malicious envy may lead individuals to disparage or denigrate the envied^3,7^. Therefore, there are some differences in one’s cognition and behavioral tendencies depending on which sort of envy one is experiencing.

Meanwhile, some researchers have proposed that values, as the core driving force of individuals^8^, are also internal factors affecting emotions^9^. Labor values, which stem from one’s basic general views and attitude, determine one’s judgment and behavioral orientation towards labor. According to Chao and Wang^10^, labor values are made up of five dimensions: honest labor value, equality status of labor value, cherishing labor value, loving labor value, and distribution value according to work. Zhou and Liu^11^ proposed that individuals with positive labor values can cherish both their own and others’ labor achievements, while deeply understanding hard-won labor achievements and identifying with others’ deserved accomplishments achieved through their own labor. This relates closely to the key factor of distinguishing benign envy from malicious envy, that is, deservedness. Individuals with positive labor values may achieve their goals through down-to-earth, diligent work effort if they think the efforts are deserved, whereas individuals with negative labor values may avoid putting in effort while working because they believe that hardship is undeserved. Therefore, it may be that labor values positively predict benign envy and negatively predict malicious envy.

Mindfulness refers to the ability of consciously keeping one’s attention focused on their present experience without judgment^12^, and is featured by enhanced levels of openness, curiosity, and acceptance about one’s experiences^13,14^. Studies have found that mindfulness can decrease the frequency and intensity of negative emotions^14^ such as anxiety and depression^15^. The internal mechanism of mindfulness can be explained by the mindfulness reperceiving model. That is, high mindfulness can help individuals identify the content of consciousness and allow them to view an experience occurring in the present moment from a clearer and more objective perspective^16^. This increases one’s cognitive and emotional freedom, which helps them avoid being troubled by negative experiences, thereby avoiding negative emotions^17^. As two negative emotions of different qualities, benign envy and malicious envy may then also be affected by mindfulness.

Mindfulness is essentially a state of consciousness whose core components include intention, attention, and attitude^16^. It is featured by a separation of the self from one’s consciousness, allowing an individual to accept the present moment without judgement, even if the situation is unfavorable^12,18^. Therefore, mindfulness is closely related to positive cognition and value judgment. Individuals with positive labor values embrace the dimensions of loving labor, willingly accepting labor, paying attention to the labor process, and shifting from the passive concept of “having to work” to the active intention of “being willing to work”^11^. This sort of positive cognition or value judgment may increase one’s willingness to accept the present and improve individual mindfulness levels. Therefore, it is reasonable to hypothesize that labor values may influence both benign and malicious envy through the mediating role of mindfulness.

### The present study

The current study explored the impact of labor values on benign envy and malicious envy, as well as the mediating role of mindfulness between labor values and benign and malicious envy in the context of Chinese culture. Furthermore, a longitudinal study design was adopted in order to explore both the immediate and delayed effects at play. The specific hypotheses were as follows:

Hypothesis 1 (H1)

Labor values positively predicts benign envy and negatively predicts malicious envy;

Hypothesis 2 (H2)

Mindfulness mediates the effect of labor values on both benign envy and malicious envy;

Hypothesis 3 (H3)

The effect and mechanism of labor values on benign envy and malicious envy is not only immediate but also delayed.

## Method

### Participants

Using a cluster sampling method, one primary school (grades 4, 5, and 6), two junior middle schools (grades 7 and 8), and one senior high school (grade 10) in a city in southeast China were selected, and a total of 2,928 participants were selected to participate by completing two surveys with an eight months’ interval between the two test time points. At Time 1 (T1), 2928 questionnaires were sent out, but only 2749 valid questionnaires were returned, making an effective recovery of 93.89%. At Time 2 (T2), due to some participant attrition (due to having a day off, suspension from school, or transferring to another school; furthermore, some questionnaires were invalid due to a series of missed questions or the same answer repeated on all pages), a total of 2363 valid questionnaires were obtained, resulting in 85.95% effective participation in both surveys. A *t*-test was conducted on the full total of participants at both time points (*n* = 2363) compared to those who only took part at T1 (*n* = 386) and no significant differences were found in labor values, mindfulness, benign envy, and malicious envy between the two. Therefore, the results of the data analysis comprising both time points were deemed to be valid. Therefore, the data of the 2,363 participants were analyzed (See Table 1, *M*_age_ = 12.46, *SD* = 1.98, 44.99% female). Of these participants, 1,179 were primary school students, 584 were junior middle school students, and 600 were senior high school students. All participants and their parents had given their informed consent to take part in the study, and had been told they had the right to withdraw freely at any point. Furthermore, they were told their personal information and results would be kept strictly confidential. All participants received a gift (about $1) as compensation for their participation. The Medical Ethics Committee of Hunan Normal University (the ethical approval ID: 2021–051) and the junior middle schools’ administration approved the research protocol. The study was conducted according to the principles in the Declaration of Helsinki. The research was performed in accordance with relevant guidelines and regulations.

**Table 1:** Demographic characteristics of the sample who finished both two tests (T1, T2, N=2363).

Variable	N	Percent	M	SD
Gender				
male	1300	55.01%		
female	1063	44.99%		
Age			12.46	1.98
9–11	893	37.8%		
12–14	916	39.8%		
15–17	554	23.4%		
Grade			6.9	2.16
4–6 (primary school students)	1179	49.90%		
7–8 (junior middle school students)	584	24.71%		
10 (senior high school students)	600	25.39%		

## Measurements

### The labor values scale

The Labor Values Scale was created by Chao and Wang^10^ for use in the Chinese cultural context, and has been shown to be applicable to students in the senior grades of primary school, middle school, and high school. The scale measures five dimensions: honest labor value, equality status of labor value, cherishing labor value, loving labor value, and distribution value according to work. Due to the low data factor-loading degree of “distribution according to work” in the current study, this dimension was excluded. A total of 12 items were selected for use from the original scale, with each item ranked on a five-point scale ranging from 1 (completely agree) to 5 (completely agree). Examples of the items are, “I enjoy the process of working” and “I respect workers of all professions equally, whether they are cleaners or engineers”. The higher the total score, the more positive one’s labor values. This study used Confirmatory Factor Analysis (CFA) to confirm the structure of scale, indicating that the four-factor model was acceptable (χ^2^/df = 11.32, RMSEA = 0.066, RMR = 0.045, GFI = 0.963, IFI = 0.935, TLI = 0.910). The Cronbach’s α coefficient of the scale in the current study was 0.827, the original scales’ Cronbach’s α was 0.81^10^.

### The child and adolescent mindfulness measure

The Child and Adolescent Mindfulness Scale, developed by Greco, Baer^19^, comprises 10 items, each rated on a five-point scale ranging from 1 (never) to 5 (always). A higher total score indicates a greater level of mindfulness thinking. In the present study, the Cronbach’s α coefficient for the scale was 0.829, while in the original study it was reported as 0.81^19^.

### The benign and malicious envy scale

The present study used the Benign and Malicious Envy Scale developed by Lange and Crusius^7^, which measures two subscales: benign envy and malicious envy. Each subscale is composed of five items, with each item scored on a six-point scale ranging from 1 (completely disagree) to 6 (totally agree). A prior study involving Chinese middle school students demonstrated sufficient internal reliability for the malicious and benign envy scales (Benign envy: Cronbach’s alpha = 0.73, Malicious envy: Cronbach’s alpha = 0.85)^20^. In the current study, the Cronbach’s α coefficient of benign envy was 0.723 at T1 and 0.757 at T2, and the Cronbach’s α coefficient of malicious envy was 0.790 at T1 and 0.805 at T2.

### Procedure

Each investigation at each specific time point was done as a part of a comprehensive psychological assessment. Before beginning the data collection, informed consent was obtained from the schools, the students taking part in the study and the students’ parents. Time was arranged with the schools to administer the questionnaires, at which point teachers distributed the questionnaires accompanied by the agreed-upon presentation text and explanation of the test procedures, emphasizing that all responses would remain confidential. Completion of the questionnaires at both time points took roughly 40 min. Before each investigation, the teachers in charge of each student group received 30 min’ training beforehand regarding the study procedures from professional psychological teachers.

The following inclusion criteria were employed: (1) Participation was voluntary; (2) Their school-based mental health clinician did not perceive their participation as overly disruptive; (3) Students possessing adequate literacy skills, as confirmed by experienced schoolteachers, capable of comprehending survey questions without difficulty.

### Data analysis

Stata 16 was used to merge the data from each of the two time points, then SPSS 18.0 and AMOS 17.0 were used to analyze the data and build the structural models.

### Ethics approval

Ethics approval was obtained from the Medical Ethics Committee of Hunan Normal University (the ethical approval ID: 2021-051). Written informed consent was obtained from all participants.

### Informed consent

Informed consent was obtained from all individual participants included in the study.

## Results

### Control and inspection of common method biases

The data used in this study was collected using a self-report method, which can lead to a common method bias effect^21^. To avoid this, a Harman single-factor test was used for the common method deviation test^22^, and an exploratory factor analysis was conducted on the six measured variables twice. The results showed that there were six factors with characteristic roots over one at both time points, and the variance explained by the first factor was 20.52% at T1 and 18.48% at T2, both of which were lower than the critical standard of 40%.

### Reliability and validity

Before evaluating the measurement model, it is essential to assess the questionnaire’s reliability and validity. Convergent validity was examined by confirmatory factor analysis, by examining the Composite Reliability (CR) and Average Variance Extracted (AVE). If CR is above 0.7 and AVE is over 0.5, it’s considered acceptable for the items measuring variables to be consistent^23^. In our study, all CR values exceeded 0.7, and AVE was above 0.5 except for the Labor Values Scale, where it was 0.491 (See to Table 2). This suggests that the measurement showed good internal consistency, and the reliability was acceptable.

**Table 2:** Convergent validity.

	Items	Standardized estimates	Standard error	Z-value	CR	AVE
T1 labor value	T1 CE	0.696			0.788	0.491
	T1 LV	0.745***	0.04	30.808		
	T1 EQ	0.478***	0.035	20.71		
	T1 HN	0.832***	0.038	32.515		
T1 mightfulness	T1 MF1	0.727			0.779	0.543
	T1 MF2	0.834***	0.039	29.17		
	T1 MF3	0.637***	0.038	26.798		
T1 benign envy	T1 BE1	0.943			0.759	0.623
	T1 BE2	0.598***	0.028	16.174		
T1 malicious envy	T1 ME1	0.815			0.819	0.694
	T1 ME 2	0.851***	0.029	23.217		
T2 benign envy	T2 BE 1	0.922			0.776	0.641
	T2 BE 2	0.657***	0.043	12.507		
T2 malicious envy	T2 ME 1	0.88			0.826	0.705
	T2 ME 2	0.797***	0.036	17.217		

***p < 0.001. BE: benign envy; ME: malicious envy; MF: mightfulness; HN: honest labor value; EQ: equality status of labor value; CE: cherishing labor value; LV: loving labor value; DS: distribution value according to work. The same in following tables/figures.

If the correlation between a variable and other variables is lower than the square root of the mean variance, it suggests good discriminant validity for that variable^23^. In Table 3, the bolded data represent the square root of the mean variance. Notably, these values were higher than all the other values in their respective columns. Therefore, the discriminant validity of our measurement model is deemed appropriate.

**Table 3:** Discriminant validity.

	AVE	T1 BE	T1 MF	T1 labor value	T1 ME
T1 BE	0.623	**0.789**			
T1 MF	0.543	0.171	**0.737**		
T1 labor value	0.491	0.534	0.274	**0.701**	
T1 ME	0.694	− 0.085	− 0.439	− 0.339	**0.833**

The BOLD indicate square root of the mean variance.

### Descriptive statistics and correlation analysis

Table 4 presents the mean, standard deviation, and correlation matrix of each variable at T1 and at T2. The results showed that labor values correlated positively with benign envy at both T1 (*r* = 0.43, *p* < 0.01) and T2 (*r* = 0.30, *p* < 0.01), and correlated negatively with malicious envy at both T1 (*r* = − 0.28, *p* < 0.01) and at T2 (*r* = − 0.22, *p* < 0.01). However, only a weak correlation was found between mindfulness and benign envy at T1 (*r* = 0.07, *p* < 0.01), and no correlation was found between mindfulness and benign envy at T2 (*r* = 0.03, *p* = 0.102). As for mindfulness and malicious envy, negative correlations were found at T1 (*r* = − 0.34, *p* < 0.01) and T2 (*r* = − 0.24, *p* < 0.01). Meanwhile, there was a significant positive correlation found between labor values and mindfulness (*r* = 0.17, *p* < 0.01). The correlational relationships found provided a preliminary reference value for further analysis of the relationship between the variables.

**Table 4:** Descriptive Statistics and Correlation Coefficients for Each Variable.

Variable	1	2	3	4	5	6
1. Labor values at T1	1					
2. Mindfulness at T1	0.17**	1				
3. Benign Envy at T1	0.43**	0.07**	1			
4. Malicious envy at T1	− 0.28**	− 0.34**	− 0.01	1		
5. Benign envy at T2	0.30**	0.03	0.39**	− 0.06**	1	
6. Malicious envy at T2	− 0.22**	− 0.24**	− 0.05*	0.46**	0.07**	1
Mean	46.93	35.26	22.51	11.99	22.50	11.39
Standard Deviation	7.35	7.30	4.65	5.51	5.20	5.93

*p < 0.05, **p < 0.01, ***p < 0.001, T1 = Time 1, T2 = Time 2.

### The relationship between labor values and benign/malicious envy: the mediating role of mindfulness

The results of the correlation analysis showed that labor values, benign envy, malicious envy, and mindfulness were all correlated with one another, providing support for further analysis of the mediating effect. The immediate effect model (M1) was constructed using labor values at T1 as the independent variable, benign envy and malicious envy at T1 as the dependent variables, and mindfulness at T1 as the mediating variable. Then, the delayed effect model (M2) was constructed using labor values at T1 as the independent variable, benign envy and malicious envy at T2 as the dependent variables and mindfulness at T1 as the mediating variable.

The maximum likelihood estimation method was used to determine the parameters of both models. The fitting data indices of the models are shown in Table 5, and the fit was good.

**Table 5:** Fit Indices of M1 and M2.

	*χ*^*2*^	*df*	*χ*^*2*^*/df*	*RMSEA*	*SRMR*	*TLI*	*CFI*	*AIC*	*ECVI*
M1	517.248	39	13.263	0.072	0.055	0.922	0.945	593.248	0.251
M2	376.896	39	9.664	0.061	0.049	0.942	0.959	452.896	0.192

RMSEA: Root mean square error of approximation, SRMR: Standardized root mean square residual, CFI: Comparative fit index, TLI: Tucker-Lewis index, AIC: Akaike information criterion, ECVI: Expected cross-validation index, M1: the immediate effect model, M2: the delayed effect model.

**Figure 1:**
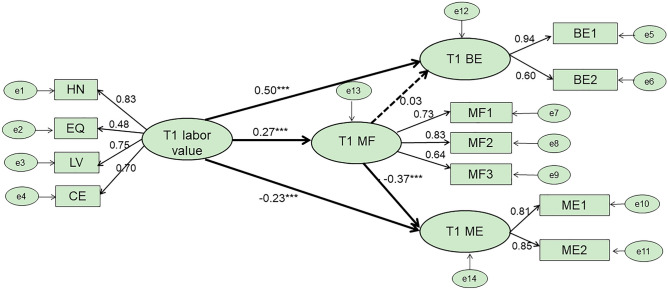
Direct and indirect effects of labor values on benign envy and malicious envy using Squares Structural Equation Modeling (Time 1).

The standard path coefficients for M1 are shown in Fig. 1. Labor values at T1 significantly positively predicted benign envy at T1 (β = 0.50, *p* < 0.001), negatively predicted malicious envy at T1 (β = − 0.23, *p* < 0.001), and positively predicted mindfulness at T1 (β = 0.27, *p* < 0.001). Meanwhile, mindfulness at T1 also significantly negatively predicted malicious envy at T1 (β = −0.37, *p* < 0.001), however mindfulness at T1 did not significantly positively predict benign envy at T1 (β = 0.03, *p* = 0.195). All potential mediating effects were further tested using the bias-corrected percentile bootstrap method. To test the mediating effect of mindfulness at T1 in the relationship between labor values at T1 and both benign and malicious envy at T1, we extracted 2,000 bootstrap samples from the original data. The results showed that mindfulness at T1 played a significant mediating role in the labor values at T1 → malicious envy at T1 pathway (β = − 0.103, 95% confidence interval [− 0.130, − 0.079]). but no significant mediating role in the labor values at T1 → benign envy at T1 pathway was found.

**Figure 2:**
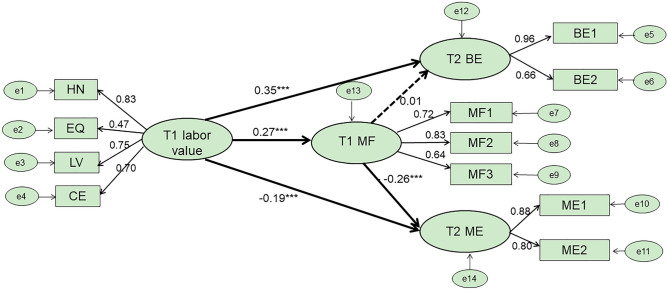
Delayed effect of labor values on benign envy and malicious envy using Squares Structural Equation Modeling (Time 2).

We then looked for a delayed effect using M2, as shown in Fig. 2, and found that labor values at T1 significantly positively predicted benign envy at T2 (β = 0.35, *p* < 0.001), significantly negatively predicted malicious envy at T2 (β = − 0.19, *p* < 0.001), and significantly positively predicted mindfulness at T1 (β = 0.27, *p* < 0.001). Meanwhile, mindfulness at T1 also significantly negatively predicted malicious envy at T2 (β = − 0.26, *p* < 0.001), however it did not significantly positively predict benign envy at T2 (β = 0.01, *p* = 0.571). To test the mediating effect of mindfulness at T1 in the relationship between labor values at T1 and benign and malicious envy at T2, we extracted 2000 bootstrap samples from the original data. The results showed that mindfulness at T1 played a significant mediating role in the labor values at T1 → malicious envy at T2 pathway (β = − 0.070, 95% confidence interval [− 0.092, − 0.052]), but no significant mediating role in the labor values at T1 → benign envy at T2 pathway was found.

### Gender differences

Cross-gender stability of M1 and M2 was explored using a multi-group structural equation comparison. The results indicated that both M1 and M2 were stable across gender, as shown in Table 6. As Chi-square test results are easily affected by sample size, we further compared the structural paths of both genders, that is, the critical ratio of difference (CRD) was used as an indicator to investigate cross-gender differences between the models. When the absolute value of CRD is greater than 1.96, significant differences exist in the pathways^24^. The results indicated that there were no significant differences in either M1 or M2 in the path coefficients of the male and female groups, as shown in Table 7.

**Table 6:** Comparison of Constrained and Unconstrained Structural Models by Gender.

	Model fit indexes								Model comparison	
	χ^2^	*df*	χ^2^/*df*	TLI	CFI	ECVI	AIC	RMSEA	Δχ^2^/*df*	*p*
M1										
Constrained M1	635.100	94	6.756	0.927	0.938	0.320	755.10	0.049		
Unconstrained M1	641.768	99	6.483	0.931	0.938	0.318	751.77	0.048	1.334	0.246
M2										
Constrained M2	456.32	94	4.854	0.949	0.956	0.244	576.32	0.040		
Unconstrained M2	464.598	99	4.693	0.951	0.956	0.243	574.60	0.040	1.655	0.142

RMSEA: Root mean square error of approximation, SRMR: Standardized root mean square residual, CFI: Comparative fit index, TLI: Tucker-Lewis index, AIC: Akaike information criterion, ECVI: Expected cross-validation index. M1: the immediate effect model, M2: the delayed effect model.

**Table 7:** Critical Ratio of Difference in Structural Path by Gender.

	LV → MF	LV → BE	LV → ME	MF → BE	MF → ME
M1	1.12	− 0.725	− 0.004	− 1.902	− 0.23
M2	1.126	− 0.922	0.805	− 0.842	− 1.144

LV: Labor values, MF: Mindfulness, BE: Benign envy, ME: Malicious envy, M1: the immediate effect model, M2: the delayed effect model.

## Discussion

Using the mindfulness reperceiving model, this study explored the mechanism of labor values on benign and malicious envy. The results supported the hypotheses that labor values can directly and significantly affect both benign envy and malicious envy. Furthermore, mindfulness was shown to play a different mediating role in the relationship between labor values and malicious envy, and the effect had both simultaneity and continuity.

This study found that labor values can negatively predict malicious envy and positively predict benign envy. The predictive effect was not only immediate but also delayed, which verified our hypotheses H1 and H3. The reason for this could be that individuals with positive labor values are more willing to believe in the idea of “no pain equals no gain”^10^. Therefore, compared to other individuals, people with positive labor values are more likely to interpret the achievements of an envied person as a reward for their effort, thus stimulating benign envy. Meanwhile, people with positive labor values are less likely to interpret the achievements of an envied person as unreservedness, thus feeling less malicious envy. People with positive labor values enjoy laboring, and believe that labor is a source of happiness, which leads to them experiencing more positive emotions at work. These positive emotions can expand their scope of attention and cognition^25^, and help individuals view others’ advantages or disadvantages through a social comparison perspective more comprehensively and objectively, thus increasing their benign envy while inhibiting their malicious envy.

Our results also showed that mindfulness played an immediate and delayed mediating role between labor values and malicious envy only, suggesting that the influence of mindfulness differs in the relationship between labor values and benign envy, which confirmed our hypotheses H2 and H3. More specifically, positive labor values were shown to predict malicious envy through mindfulness, but did not predict benign envy through mindfulness, which is consistent with the findings of Dong, Xiang^26^ in adult groups. According to the reperceiving model of mindfulness, reperception allows individuals to “jump out” of their script, so to speak, allowing them to recognize and re-evaluate the present moment from the objective perspectives of “stepping back” and “just observing”, thereby blocking negative emotions^12^. However, malicious envy emerges when individuals believe that the advantages attained by those they envy is undeserved, and when their consciousness is limited to focusing on the negative aspects of others^3,27^. Therefore, individuals with a high state of mindfulness, whose consciousness is less fixed on the negative qualities of others, will tend to respond in a more flexible way and be less affected by negative emotions^28,29^, thus avoiding malicious envy^26^. The mindfulness emotion regulation model may provide a further theoretical explanation, that a higher level of mindfulness helps individuals face emotions without jumping to either reaction or repressing. Instead, they may be more likely to fully perceive and accept all kinds of negative emotion, thereby improving their ability to consciously allocate and regulate their own emotional response by disengaging from an automatic judgment process, thus promoting the positive transformation of negative emotions^14^ and reducing malicious envy^30^. As for benign envy, although it is also a negative emotion that stems from social comparison, its intensity is much lower than that of malicious envy^5^. Therefore, the mediating role of mindfulness in the relationship between labor values and benign envy may not be significant.

The current study does have some shortcomings. First, the self-report method was used to collect data for this study, which may have led to subjective bias. Second, although the current study employed a longitudinal study design, only two time points were tracked, and the interval between them was only eight months. Future studies should increase the number of time points tracked while also expanding the time interval between them. Third, the study participants were students, so it may not be possible to generalize the findings to other demographics. The age range in the sample could be further expanded in future studies. Finally, future research should test whether there are cultural differences in the relationship between labor values, mindfulness, and both benign and malicious envy.

## Conclusion

This study explored the effect of labor values on both benign and malicious envy, as well as the mediating mechanism of mindfulness in the Chinese cultural context from the perspective of the mindfulness reperceiving model. Our findings have not only enriched current understandings of envy, but also the effect and impacts of labor values on individuals’ development of social emotions. Furthermore, as our findings may reflect cultural differences, future research should examine cross-cultural samples to confirm our findings in other cultural contexts. The complex relationship between labor values, mindfulness, and both benign and malicious envy were explored using a longitudinal study design, which showed that the immediate and delayed mediating role of mindfulness exists only in the relationship between labor values and malicious envy. These findings may provide valuable guidance on the future implementation of mindfulness as an intervention practice aimed at inhibiting malicious envy.

## Data Availability

The datasets used and/or analyzed during the current study available from the corresponding author on reasonable request.
